# Degree of Safety Against Falls Provided by 4 Different Prosthetic Knee Types in People With Transfemoral Amputation: A Retrospective Observational Study

**DOI:** 10.1093/ptj/pzab310

**Published:** 2022-01-13

**Authors:** Pierpaolo Palumbo, Pericle Randi, Serena Moscato, Angelo Davalli, Lorenzo Chiari

**Affiliations:** 1 Department of Electrical, Electronic, and Information Engineering “Guglielmo Marconi,” Alma Mater Studiorum University of Bologna, Bologna, Italy; 2 Unità operativa di medicina fisica e riabilitazione, INAIL Centro Protesti, Vigoroso di Budrio, Emilia-Romagna, Italy; 3 Area ricerca e formazione, INAIL Centro Protesti, Vigoroso di Budrio, Emilia-Romagna, Italy; 4 Health Sciences and Technologies, Interdepartmental Center for Industrial Research, Alma Mater Studiorum University of Bologna, Bologna, Italy

**Keywords:** Accidental Falls, Amputation, Balance, Knee, Prostheses and Implants

## Abstract

**Objective:**

People with transfemoral amputation have balance and mobility problems and are at high risk of falling. An adequate prosthetic prescription is essential to maximize their functional levels and enhance their quality of life. This study aimed to evaluate the degree of safety against falls offered by different prosthetic knees.

**Methods:**

A retrospective study was conducted using data from a center for prosthetic fitting and rehabilitation. Eligible individuals were adults with unilateral transfemoral amputation or knee disarticulation. The prosthetic knee models were grouped into 4 categories: locked knees, articulating mechanical knees (AMKs), fluid-controlled knees (FK), and microprocessor-controlled knees (MPK). The outcome was the number of falls experienced during inpatient rehabilitation while wearing the prosthesis. Association analyses were performed with mixed-effect Poisson models. Propensity score weighting was used to adjust causal estimates for participant confounding factors.

**Results:**

Data on 1486 hospitalizations of 815 individuals were analyzed. Most hospitalizations (77.4%) were related to individuals with amputation due to trauma. After propensity score weighting, the knee category was significantly associated with falls. People with FK had the highest rate of falling (incidence rate = 2.81 falls per 1000 patient days, 95% CI = 1.96 to 4.02). FK significantly increased the risk of falling compared with MPK (incidence rate ratio [IRR_FK-MPK_] = 2.44, 95% CI = 1.20 to 4.96). No other comparison among knee categories was significant.

**Conclusions:**

Fluid-controlled prosthetic knees expose inpatients with transfemoral amputation to higher incidence of falling than MPK during rehabilitation training.

**Impact:**

These findings can guide clinicians in the selection of safe prostheses and reduction of falls in people with transfemoral amputation during inpatient rehabilitation.

## Introduction

Major lower limb amputation (ie, amputation above the ankle joint) is a condition affecting approximately 25 new cases per 100,000 person-years.^1^^,^^2^ It may result from different etiologies, such as vascular diseases, infections, cancer, trauma, or congenital conditions. Lower limb amputation impairs balance and mobility. More than 50% of people with lower-limb amputation living in the community fall at least once a year.^3^^,^^4^ Along with common clinical co-morbidities (eg, pain, osteoarthritis, and mental disorders), lower limb amputation may severely limit the individual’s activities and social participation. An adequate prosthetic prescription is essential to minimize the disabling impact of the amputation and enhance individuals’ quality of life. Among this population, more proximal amputation levels are associated with more severe balance and mobility problems and higher fall risk.^3^ Amputations above the knee (knee or hip disarticulation and trans-femoral amputation) represent more than 25% of lower limb amputations.^5^

A wide variety of prosthetic knees is available for those individuals, varying significantly in terms of design, functionalities, and cost.^6–9^ Stability and safety against stumbles and falls are achieved in different prosthetic knee designs utilizing different engineering mechanisms.^10^ Locked knees (LKs) automatically lock when the knee is fully extended and can be unlocked only manually, that is, when the individual has to sit down.^10^ They achieve safety by avoiding knee failure as a possible cause of falling. Among articulating (ie, nonlocked) mechanical knees (AMKs), weight-activated stance-control knees control stability during stance through a brake activated by body weight when the knee is fully extended.^10–12^ Polycentric knees—a feature often present in AMKs—achieve stability during stance and ease of flexion during swing by utilizing a variable instantaneous center of rotation between thigh and shank, which moves back and forth during the gait cycle. Fluid-controlled knees (FKs) use pneumatic or hydraulic circuits to attain resistance to knee flexion and extension that changes during the gait cycle and accommodate different walking speeds.^10^^,^^13^ Microprocessor-controlled knees (MPKs) regulate knee resistance using a microprocessor, which processes signals from force and motion sensors (eg, strain gauges and inertial sensors) embedded in the prosthetic knee and sends a control signal to actuating parts (eg, solenoid valves).^14–16^ Evidence about the safety of the different prosthetic designs is essential in several contexts: in clinics to guide better prosthesis prescription, in public health to inform rational and equitable reimbursement criteria, and in engineering to inform better designs.

Although some studies have investigated risk factors for falls in people with lower-limb amputations,^17–19^ there remains a knowledge gap about the possible role played by the prosthesis as a contributing cause of falls. Blumetritt et al set up a gait perturbation protocol to evaluate the safety potential of prosthetic knees in a laboratory environment.^20^ The protocol was applied to test 8 knee models on 18 individuals. They showed that the C-Leg (an MPK model) offers better stability to gait perturbations than Mauch SNS and 3R80 (2 FK models with control mechanisms of flexion resistance, based respectively on knee extension and loading) and, similarly, that the C-Leg 4 is more stable than 4 other MPK models (Hybrid Knee, Adaptive 2, Rheo Knee, and Rheo Knee XC).^20–22^ However, the predictive ability of laboratory-based fall simulation protocols has never been validated on the incidence of real-world falls. In free-living conditions, safety against falls has been investigated in small pre-post or crossover studies by Kahle et al^23^ (19 participants), Wong et al^24^ (8 participants), Hafner and Smith^25^ (17 participants), and Fuenzalida Squella et al^26^ (13 participants). These authors found that MPK (or some specific models thereof) may protect against falls better than non-MPK (NMPK). Although supported by clinical reasoning, this evidence was judged to be weak in systematic reviews for clinical practice guidelines.^27^^,^^28^ Significant limitations are the small sample size of enrolled participants and the potential conflict of interest of sponsoring companies. Meanwhile, national surveillance programs for adverse events related to medical devices (eg, as foreseen by the EU Regulation on Medical Devices) do not monitor falls occurring while wearing lower-limb prostheses.

In this study, we addressed this knowledge gap by assessing the protection offered by different prosthetic knee designs against falls during inpatient rehabilitation. This study was conducted within the framework of the MOTU project (“protesi robotica di arto inferiore con sMart sOcket ed inTerfaccia bidirezionale per ampUtati di arto inferiore”).^29^

## Methods

### Study Design

We performed a retrospective, observational study using data from INAIL Prosthesis Centre in Budrio, Italy. The INAIL Prosthesis Centre encompasses rehabilitation departments and orthopedic laboratories assisting every year more than 1500 individuals with amputations of upper or lower limbs due to on-the-job injuries and other etiologies.

We defined as eligible all hospitalizations of individuals with unilateral trans-femoral amputation or knee-disarticulation, aged over 18 years, having been admitted as an inpatient at INAIL Prosthesis Centre between 2011 and 2017. We assessed prosthetic knee safety by considering falls experienced during the hospitalization while wearing the prosthesis as the outcome.

We enrolled only hospitalizations with signed informed consent from the patient for data treatment for research purposes. The study was approved by the Ethics Committee “Area Vasta Emilia Centro” (ref. MOTU 18088, CE AVEC n 380/2018/OSS/AUSLBO) and complies with the Declaration of Helsinki.

### Clinical Pathway

After the surgical amputation, individuals were first visited by a multidisciplinary team, which indicated the prosthetic components and the goal of the rehabilitation treatment. The multidisciplinary team was composed of professionals in clinical, technical, and psychosocial areas (namely, a physiatrist, an orthopedist, a nurse, a physical therapist, a prosthetist, an engineer, a psychiatrist, and a social worker). They chose the prosthetic components and the rehabilitation goal based on their experience, considering the individual’s psychophysical state, the biomechanical specificities of the amputation, and the individual’s living environment, lifestyle, personal expectations, and financial constraints. Later, the participants were admitted to the center.

During the admission visit, a physiatrist examined the participant’s general clinical conditions and their residual limb and confirmed or updated the goal of the rehabilitation training. A nurse measured the participant’s height and weight without a prosthesis, prepared a medication plan, and administered the Barthel Index^30^ for functional independence and the Morse Scale^31^ (in use until 2017) for fall risk. During admission, the participant also received a first prosthesis, used during the whole hospitalization at the center. A prosthetist performed prosthesis fitting and alignment according to the manufacturer’s indications and the participant’s specific needs. All prosthetists were qualified as certified prosthetist orthotists. Particular care was given to socket adaptation, with continuous refinements, if needed, during the rehabilitation period to guarantee the best compliance, prosthesis control, and comfort. The rehabilitation treatment was tailored according to the participant’s specific abilities, needs, and rehabilitation goals. It included exercises to improve range of motion and muscle strength as well as treatments to alleviate phantom limb pain.

After receiving primary postamputation rehabilitation, some participants returned to the center for additional rehabilitation after the substitution or significant revision of the prosthesis or its main components (namely the socket, the knee, or the foot). When there was a change from NMPK to MPK, the participant underwent a rehabilitation program with exercises specific to this new knee category. Otherwise, hospitalizations were limited to the time necessary for substituting or modifying the prosthesis components, and the rehabilitation program included general exercises that aimed to correct gait abnormalities and improve prosthesis usage.

All falls experienced by the participants at the center were recorded by the health staff using dedicated forms (Suppl. Figs. 1 and 2), as indicated by the Italian Ministry of Health’s recommendation for preventing and managing falls in health care facilities.^32^ Contextual information about falls was also collected, including whether the participant was wearing the prosthesis during the fall. Both in the prosthesis center and for the purposes of this study, a fall was defined as a sudden, unintentional, and unexpected descent from upright, seated, or clinostatic position.^32^

### Data Retrieval

We retrieved data on falls, prosthetic knees, and risk factors for falls. Risk factors for falls were identified from the literature about people with a lower-limb amputation^17^^,^^18^ or older adults.^33^ Data were retrieved from participants’ electronic health records, except participant consent for data treatment, the Barthel Index, and the Morse Scale, which were derived from paper-based medical records.

We classified prosthetic knees into 4 categories: (1) prosthetic knees used in locked configuration during walking (LK); (2) AMKs without fluid control (AMK), (3) FK, that is, non-electronic knees with hydraulic or pneumatic control; and (4) electronic knees (MPK) (Suppl. Tab. 1). Although no standard classification system exists for prosthetic knees, this categorization reflects the main functional principles of prosthetic knees available on the market and is in line with practitioners’ manuals.^6^ Information on the prosthetic knee used during each hospitalization was determined by looking at the most recent prosthesis provided prior to or during the admission. We excluded prostheses generally used for bathing (Otto Bock HealthCare GmbH, Duderstadt, Germany and Aulie Devices Inc., Bastrop, TX, USA). When 2 prosthetic knees were provided during the same hospitalization, we categorized the participants based on his/her highest category knee (LK < AMK < FK < MPK), considering the other as the back-up prosthesis.

We mapped drug names to their Anatomical Therapeutic Chemical code^34^ using publicly available tables from the Italian Drug Agency.^35^^,^^36^ We counted the number of drugs and identified the use of antipsychotics (Anatomical Therapeutic Chemical code N05A), antidepressants (N06A), benzodiazepines (N03AE, N05BA, N05CD, N05CF), loop diuretics (C03C), beta-blocking agents (C07), opioids (N02A), and antiepileptics (N03). We included these classes of drugs as well as polypharmacy because they are considered distinct risk factors for falls.^33^^,^^37–39^ The number of comorbidities was estimated by counting the number of fields filled with pathological annotations in the electronic health record section dedicated to the physical examination. We excluded those annotations indicating normal physiological functioning and the trans-femoral amputation. The rehabilitation treatment goal was codified into 4 classes: walking with a walker, walking with 2 crutches, walking with a cane or crutch, and unassisted walking. Three third-party payers were identified: the Italian Local Health Services (ASL, part of the Italian National Health System), INAIL insurance for on-the-job injuries, and participant out-of-pocket expense (private).

The anonymized data that support the findings of this study are openly available in Figshare (https://doi.org/10.6084/m9.figshare.12458225).

### Statistical Analyses

#### Participants’ Risk Factors for Falls

The association between participants’ characteristics and falls while wearing the prosthesis was assessed using mixed-effect Poisson models (function glmer, R package lme4^40^). Multiple stays related to the same participant were accounted for by a common random intercept. Different exposure times were modeled adding the logarithm of the length of stay as an offset term. Statistical significance was determined with a Wald chi-square test (function ANOVA, R package car^41^) using a threshold α = .05.

#### Propensity Score Model

To adjust the association between knee category and falls, we calculated a multivariate propensity score as the probability of receiving the assigned prosthesis, conditional on baseline participant characteristics that could act as confounding factors. Adjusting the association between 2 variables of interest using propensity score weighting is a way to estimate causal effects in observational research.^42^ The propensity score was estimated fitting a generalized boosted regression model (R package twang^43^) for average treatment effects.^44^ Following indications from methodological literature,^45^^,^^46^ we included in the model all participants’ characteristics that we identified as significant risk factors for falls that occurred while wearing the prosthesis. Missing values on generalized boosted regression model covariates were managed with simple imputation (R package mice^47^) as in Coffman et al.^48^ The ability of the propensity score to balance the 4 knee category groups were evaluated calculating the mean effect size on each participant’s characteristic, that is, the mean of the absolute standardized difference between prosthetic groups and population means.^44^

#### Association of Knee Category With Falls

The unadjusted association between the prosthetic knee category and the number of falls that occurred during the hospitalization while wearing the prosthesis was studied with mixed-effect Poisson models, as described for participants’ risk factor for falls (function glmer, R package lme4^40^). The association between knee category and falls was also adjusted with multivariate propensity score weighting.^42^^,^^44^^,^^49^^,^^50^ Incidence rates (IRs) were calculated marginalizing over the random intercept,^51^ and their CI was derived with bootstrap. Statistical significance for the prosthesis category was assessed with the Wald chi-square test. Post-hoc, Tukey’s pairwise comparisons between knee categories, controlling for family-wise error rate, were performed with function glht from the R package multcomp.^52^ The incidence rate ratio (IRR) was used to quantify the relative risk between knee category pairs.

All code used for running the statistical analyses is available from the authors on request.

### Role of the Funding Source

P. Randi and A. Davilli are coauthors of this work and employees of the funder of this study, the Istituto Nazionale Assicurazione Infortuni sul Lavoro (INAIL). They have substantially contributed to the study design, data collection, and discussion of the results.

## Results

### Sample

We identified 2429 eligible hospitalizations relating to 1235 individuals. After excluding individuals who did not attend the rehabilitation gym, whose paper-based medical record was not available, or who did not provide their consent for data treatment for research purposes, we kept in the study 1486 hospitalizations of 815 individuals. Men accounted for 91.0% of all hospitalizations (1352 hospitalizations relating to 718 patients); the age range spanned from 18 to 91 years (mean 58.1, SD 14.7 years). Most hospitalizations (77.4%, 739) were related to individuals with amputation due to trauma. Approximately one-quarter of the stays (22.2%, 326) were for rehabilitation after the first prosthetic provision.

The most frequent prosthetic knee category was MPK (40.4%, 583), followed by FK (34.5%, 498), LK (17.4%, 251), and AMK (7.8%, 113). Statistics on knee models are available in Supplementary Table 1. Other descriptive statistics for participants’ characteristics and their distribution according to prosthetic knee category are shown in Table 1.

**Table 1:** Descriptive Statistics on the Hospitalizations for the Whole Sample and Split Per Prosthetic Knee Category^*a*^

**Characteristics**	**All**					
**Variable**		**Missing Data (%)**	**LK**	**AMK**	**FK**	**MPK**
Age	58.2 (14.7)	0	70.9 (11.6)	60.7 (17.2)	54.8 (14)	55.3 (12.7)
Sex		0				
Female	134 (9%)		59 (23.5%)	14 (12.4%)	49 (9.8%)	8 (1.4%)
Male	1352 (91%)		192 (76.5%)	99 (87.6%)	449 (90.2%)	575 (98.6%)
Weight, kg	77.3 (14.3)	31 (2.1%)	72 (14.9)	74.5 (14.1)	78.1 (14.3)	79.2 (13.4)
Height, m	1.7 (0.1)	22 (1.4%)	1.7 (0.1)	1.7 (0.1)	1.7 (0.1)	1.7 (0.1)
Reason for rehabilitation training		15 (1.01%)				
First prosthetic provision	326 (22.2%)		114 (45.4%)	23 (20.5%)	166 (33.8%)	16 (2.8%)
Prosthesis renewal	1145 (77.8%)		137 (54.6%)	89 (79.5%)	325 (66.2%)	561 (97.2%)
Length of stay, d	21.7 (17.4)	2 (0.1%)	26.9 (16.1)	20.6 (13.8)	23.9 (15.6)	17.9 (19.2)
Goal of rehabilitation training		109 (7.3%)				
Unassisted gait	718 (52.1%)		24 (9.7%)	46 (41.8%)	222 (46.6%)	405 (79.7%)
Walking with cane or crutch	466 (33.8%)		106 (42.7%)	57 (51.8%)	200 (42%)	95 (18.7%)
Walking with 2 crutches	139 (10.1%)		73 (29.4%)	7 (6.4%)	47 (9.9%)	7 (1.4%)
Walking with a walker	54 (3.9%)		45 (18.1%)	0 (0%)	7 (1.5%)	1 (0.2%)
Third-party payer		2 (0.1%)				
ASL	321 (21.6%)		112 (44.6%)	46 (40.7%)	139 (27.9%)	10 (1.7%)
INAIL	1096 (73.9%)		110 (43.8%)	63 (55.8%)	332 (66.7%)	566 (97.1%)
Private	67 (4.5%)		29 (11.6%)	4 (3.5%)	27 (5.4%)	7 (1.2%)
Time from amputation, mo	204.8 (207.9)	222 (14.9%)	154.6 (209.9)	328.4 (244.3)	139.4 (190.2)	264.9 (185.9)
Amputation side		6 (0.4%)				
Left	781 (52.8%)		108 (43.2%)	54 (48.2%)	274 (55.1%)	329 (56.5%)
Right	699 (47.2%)		142 (56.8%)	58 (51.8%)	223 (44.9%)	253 (43.5%)
Amputation cause		531 (35.7%)				
Cancer	32 (3.4%)		9 (5.1%)	3 (4.3%)	19 (5.3%)	1 (0.3%)
Congenital	12 (1.3%)		1 (0.6%)	4 (5.7%)	1 (0.3%)	0 (0%)
Infectious disease	25 (2.6%)		6 (3.4%)	4 (5.7%)	13 (3.6%)	1 (0.3%)
Traumatic	739 (77.4%)		68 (38.2%)	50 (71.4%)	286 (79.4%)	322 (98.8%)
Vascular disease	147 (15.4%)		94 (52.8%)	9 (12.9%)	41 (11.4%)	2 (0.6%)
No. of comorbidities	5.3 (2.2)	2 (0.1%)	6.9 (2)	5.2 (2)	5.3 (2.2)	4.7 (2)
No. of drugs	3.5 (4)	0	6.6 (4.6)	3.7 (4.3)	3.3 (3.9)	2.4 (3)
Use of antipsychotics	32 (2.2%)	0	8 (3.2%)	1 (0.9%)	19 (3.8%)	4 (0.7%)
Use of antidepressants	128 (8.6%)	0	40 (15.9%)	4 (3.5%)	44 (8.8%)	36 (6.2%)
Use of benzodiazepines	45 (3%)	0	12 (4.8%)	4 (3.5%)	14 (2.8%)	14 (2.4%)
Use of loop diuretics	86 (5.8%)	0	51 (20.3%)	4 (3.5%)	18 (3.6%)	11 (1.9%)
Use of beta-blocking agents	234 (15.7%)	0	74 (29.5%)	15 (13.3%)	66 (13.3%)	75 (12.9%)
Use of opioids	83 (5.6%)	0	16 (6.4%)	6 (5.3%)	38 (7.6%)	22 (3.8%)
Use of antiepileptics	145 (9.8%)	0	40 (15.9%)	4 (3.5%)	73 (14.7%)	27 (4.6%)
Morse Scale	39.1 (14.4)	438 (29.5%)	47.4 (16.8)	40.3 (13.4)	39 (14.2)	36 (12.2)
Barthel Index	92.6 (12.2)	550 (37.0%)	80.6 (17.7)	92.1 (9.9)	91.9 (10.3)	99.4 (2.8)
Prosthetic knee category		41 (2.8%)	251 (17.4%)	113 (7.8%)	498 (34.5%)	583 (40.4%)

^*^a^*^Continuous variables are described as mean (SD); categorical variables as frequencies (%). AMK = articulating mechanical knee; ASL = Italian Local Health Services; FK = fluid-controlled knee; INAIL = National Insurance for On-The-Job Injuries: LK = locked knee; MPK = microprocessor-controlled knee.

One-hundred nine (109) falls occurred in 88 individuals (94 hospitalizations) during 32,213 inpatient-days, resulting in a marginal IR for any fall of 3.35 (95% CI = [2.25 to 4.45]) falls per 1000 patient-days, as estimated by the mixed-effect Poisson model. Information on worn prosthesis was missing on 16 falls, resulting in 93 falls available for further analyses. Most of these falls (73.1%, 68 falls) occurred while the participant was wearing the prosthesis (IR = 2.08, 95% CI = [1.19 to 3.30]).

### Participants’ Risk Factors for Falls and Propensity Score Model

The association between participants’ characteristics and falls wearing the prosthesis is shown in Table 2. Significant risk factors for falls with a prosthesis are reason for rehabilitation training (IRR for prosthetic renewal vs first prosthetic provision = 0.47, 95% CI = [0.28 to 0.78]), time from amputation (IRR for 1-unit increase in the logarithmic scale = 0.78, 95% CI = [0.66 to 0.91]), cause of the amputation (IRR with respect to cancer: congenital = 0.57, 95% CI = [0.05 to 6.17]; infectious disease = 1.29, 95% CI = [0.32 to 5.25]; traumatic = 0.43, 95% CI = [0.15 to 1.29]; vascular disease = 0.22, 95% CI = [0.06 to 0.85]), use of antidepressants (IRR = 3.25, 95% CI = [1.78 to 5.94]), and use of antiepileptics (IRR = 2.74, 95% CI = [1.56 to 4.82]). No association was found for age or sex.

**Table 2:** Associations Between Patients’ Characteristics With Falls With a Prosthesis^*a*^

**Characteristic**	**Falls With Prosthesis**		
**Variable**	**IRR**	**95% CI**	***P***
Age (for 1-year increase)	0.99	(0.97 to 1)	.12
Sex			
Male	1.58	(0.6 to 4.16)	.36
Female	1		
Weight (for 1-kg increase)	1.00	(0.98 to 1.02)	.90
Height (for 1-m increase)	7.46	(0.28 to 201.2)	.23
Reason for rehabilitation training			
Prosthesis renewal	0.47	(0.28 to 0.78)	**.004**
First prosthetic provision	1		
Goal of rehabilitation training			
Unassisted gait	0.63	(0.36 to 1.09)	.19
Walking with 2 crutches	0.60	(0.24 to 1.51)	
Walking with walker	0.23	(0.03 to 1.73)	
Walking with 1 cane or crutch	1		
Third-party payer			
INAIL	0.71	(0.4 to 1.26)	.45
Private	1.01	(0.36 to 2.87)	
ASL	1		
Time from amputation (for 1-unit increase in logarithmic scale)	0.78	(0.66 to 0.91)	**.002**
Amputation side			
Right	0.65	(0.37 to 1.12)	.12
Left	1		
Amputation cause			
Congenital	0.57	(0.05 to 6.17)	**.05**
Infectious disease	1.29	(0.32 to 5.25)	
Traumatic	0.43	(0.15 to 1.29)	
Vascular disease	0.22	(0.06 to 0.85)	
Cancer	1		
No. of comorbidities	1.09	(0.98 to 1.22)	.13
No. of drugs	1.04	(0.99 to 1.1)	.13
Use of antipsychotics	1.95	(0.62 to 6.1)	.25
Use of antidepressants	3.25	(1.78 to 5.94)	**.0001**
Use of benzodiazepines	0.56	(0.13 to 2.47)	.45
Use of loop diuretics	1.56	(0.68 to 3.55)	.29
Use of beta-blocking agents	0.81	(0.39 to 1.71)	.59
Use of opioids	1.20	(0.52 to 2.78)	.67
Use of antiepileptics	2.74	(1.56 to 4.82)	**.0005**
Morse Scale	1.01	(0.99 to 1.03)	.39
Barthel Index	0.99	(0.97 to 1.02)	.57

^*^a^*^IRRs are estimated with mixed-effect Poisson models. Significant *P* values are highlighted in bold. IRRs = incidence rate ratios. Significant *P* values are highlighted in bold.

The propensity score model was fitted with the significant participants’ risk factors for falls: the reason for rehabilitation training (prosthetic renewal vs first prosthetic provision), time from amputation, cause of the amputation, use of antidepressants, and use of antiepileptics. The maximum mean effect size after propensity score weighting over these variables was 0.102 (Suppl. Tab. 2), signifying good balance.

### Association of Knee Categories With Falls

**Figure 1:**
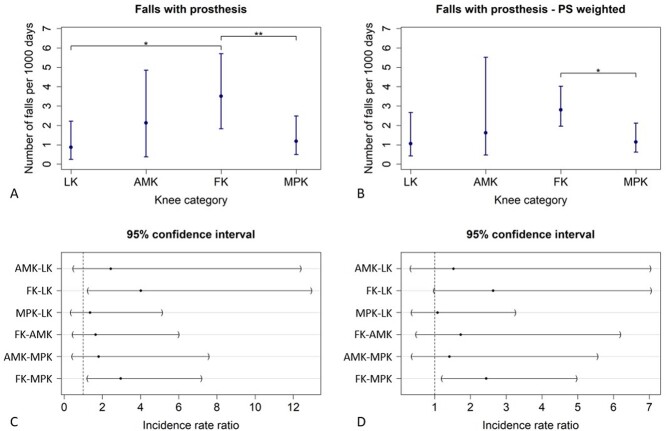
Falls incidence rates (IRs) per knee category (A–B) and incidence rate ratios (IRRs) per knee category pairs (C–D). IRs and IRRs are calculated for falls that occurred while the individual was wearing the prosthesis, without adjusting for confounding effects (A, C) and after propensity score (PS) weighting (B, D). Horizontal lines marked with asterisks indicate statistically significant differences between couples of knee categories. *^a^P* < .05, *^b^P* < .001. Error bars indicate 95% CI.

Unadjusted and propensity score–weighted IRs and IRRs of prosthetic knee categories for falls with a prosthesis are shown in Figure 1 and Table 3.

**Table 3:** Hospitalizations, Patients, Time at Risk, and Falls According to Knee Category^*a*^

	**Knee Category**					
	**LK**	**AMK**	**FK**	**MPK**	**NA**	**All**
Hospitalizations (%)	251 (16.9%)	113 (7.6%)	498 (33.5%)	583 (39.2%)	41 (2.8%)	1486
Patients	189	83	334	260	30	815
Hospitalization days	6760	2332	11,889	10,453	779	32,213
Falls with prosthesis	6	5	41	14	2	68
IR (95% CI) for falls with prosthesis per 1000 patient days – unadjusted	0.87 (0.25 to 2.22)	2.13 (0.38 to 4.86)	3.51 (1.83 to 5.71)	1.19 (0.50 to 2.49)		2.08 (1.19 to 3.30)
IR (95% CI) for falls with prosthesis per 1000 patient days – PS-weighted	1.07 (0.43 to 2.67)	1.63 (0.48 to 5.52)	2.81 (1.96 to 4.02)	1.15 (0.62 to 2.12)		

^*^a^*^AMK = articulating mechanical knee; FK = fluid-controlled knee; IR = incidence rate; LK = locked knee; MPK = microprocessor-controlled knee; NA = not available; PS = propensity score.

The knee category was significantly associated with falls with a prosthesis (*P* = .001). In particular, FKs expose patients to a higher fall rate than LKs (IRR_FK-LK_ = 4.01, 95% CI = [1.24 to 12.94], *P* = .013) and MPK (IRR_FK-MPK_ = 2.96, 95% CI = [1.22 to 7.18], *P* = .009).

A statistically significant association (*P* = .038) between knee category and falls was conserved after propensity score weighting. Looking at pairwise comparisons between knee categories, only the IRR between FK and MPK was statistically significant (IRR_MPK-FK_ = 2.44, 95% CI = [1.20 to 4.96], *P* = .014).

**Figure 2:**
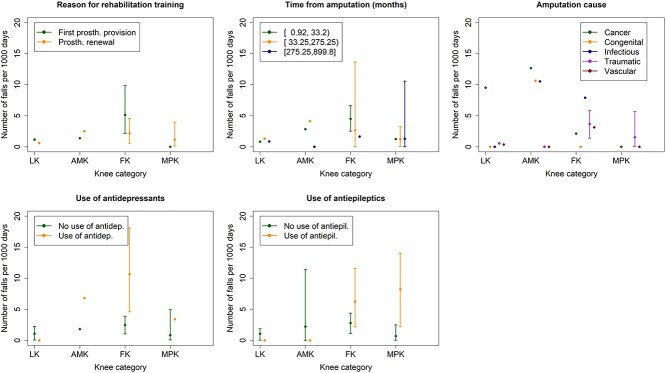
Incidence rates (IRs) for falls occurred while wearing the prosthesis, according to knee category and patients’ risk factors. Error bars indicate 95%CIs. IRs and CIs are estimated with mixed-effect Poisson models on groups with at least 5 falls. For the other groups, crude IRs are reported without CI.

Figure 2 shows fall incidence according to knee category and participants’ risk factors.

## Discussion

This study shows that FK was associated with a significantly increased risk of falling compared with MPK. This result was obtained after adjusting for confounding factors with a propensity score approach, showing that knee category may be causally related to falls.

These results are in line with other literature findings that have compared MPK with NMPK in terms of protection against falls, stumbles, or perceived stability.^11^^,^^23–26^^,^^53^ In further detail, we found that among NMPK designs, FK was associated with the highest fall incidence. Safety of LK is expected by design, though at the expense of a nonnatural gait. AMK represents a heterogeneous category encompassing, for example, weight-activated stance-control knees and polycentric knees.

We observed (Fig. 2) that the fall IR on FK was higher in patients at their first prosthetic outfitting than at prosthesis renewal. This finding may be the effect of 2 interacting factors. First, patients in rehabilitation for their first prosthesis (hence, after a short time from amputation) are at increased risk of falling (Tab. 2), in agreement with previous literature.^17^ Secondly, FK may be too complex to use at first provision and may impose a higher cognitive burden than MPK.^7^^,^^25^^,^^54^ The possibility offered by FK to regulate the mechanical impedance of the knee joint is similar to the one offered by MPK. However, MPK provide safety mechanisms that allow blocking the knee when an incipient fall is detected. Thus, FK may have similar complexity but less safety.

We noted that the use of antidepressants and antiepileptics were risk factor for falls with the largest effect sizes (antidepressants: IRR = 3.25, 95% CI = 1.78 to 5.95; antiepileptics: IRR = 2.74, 95% CI = 1.56 to 4.82; Tab. 2) compared with both the effect of other patients’ risk factors and the effect of knee category (Fig. 2). Because these drug classes are frequently used in this population for treating depression and neurogenic pain (in our dataset, frequencies of use of antidepressants and antiepileptics were 8.6% and 9.8%, respectively), particular attention must be paid to those patients under risk-associated pharmacological treatments, and future research should investigate the best prosthetic options for them.

Although falls in people with lower-limb amputations are considered major health events and receive the attention of public health researchers, current health technology assessments rely on a limited knowledge base.^55–57^ The present study assessed the safety of prosthetic knees grouped into 4 categories. The evidence was based on more than 30 different knee models (Suppl. Tab. 1) worn by 815 individuals during 1486 hospitalizations. The individuals experienced 68 falls occurring during the hospitalization while wearing their prosthesis. We believe that focusing on falls that unintentionally occurred within a rehabilitation setting has provided evidence that complements the findings from studies based on falls induced on experimental perturbation platforms.

Nonetheless, rehabilitation wards have some particular properties that make them different from other settings when it comes to the risk of falling. On the one hand, a rehabilitation ward is expected to contain fewer environmental hazards than one’s home or the community. On the other hand, it may be perceived as less familiar, thus generating disorientation. Furthermore, individuals undergoing rehabilitation generally have less acquaintance with their prosthesis and reduced balance control. Consequently, as their risk factors for falls change according to setting and time since amputation,^3^^,^^17^^,^^58^ the safety profile of different prostheses may also change. Therefore, the generalizability of our findings to people with amputation living in the community must still be determined.

Individuals at their first rehabilitation training differ in several aspects from those returning to the center for prosthesis renewal. For example, they have lower baseline mobility and functional levels and, consequently, larger margins for improving during their first rehabilitation. Interesting insights may be gained by performing a subgroup analysis on individuals at first provision. As an exploratory analysis, we have plotted the fall IR on different strata of individuals’ risk factors for falls per knee category (Fig. 2). However, we did not carry out any subgroup analysis or any testing for interactions between patient risk factors and knee category due to limits in the sample size of the dataset. Because subgroup analyses could not be performed, we cannot conclude whether there are differential effects of different knee types on individuals with different clinical profiles. The aim of obtaining evidence for this heterogeneity of treatment effect has yet to be achieved^59^ and would guide personalized prescription of lower-limb prostheses. Larger sample sizes could be obtained establishing registries for individuals with lower limb amputation or collecting data from multiple clinical sites over a shared protocol. Mobile technologies could enable the patients’ ecological observation outside the rehabilitation setting during their daily lives. Further research to address some of these topics is ongoing.

In this study, we estimated the fall IR with mixed-effect Poisson regressions. These models allowed modeling hospitalizations with different lengths of stay and multiple hospitalizations relative to the same individuals. Some authors have suggested modeling fall counts with negative binomial regression or zero-inflated Poisson models to accommodate overdispersion and excess zero-count data.^60^^,^^61^ However, after a sensitivity analysis, on our data the mixed-effect Poisson model proved to be the best choice in terms of Akaike and Bayesian information criteria (data not shown). This choice also allowed us to interpret the fitted parameters in terms of IRs and IRRs, which is less straightforward with zero-inflated models.

The main limitations of this study are related to its observational design, the absence of physical performance measures, the high proportion of missing data, and the patient mix representativeness.

The observational design of the study hinders the inference of causal relationships from statistical associations. However, we adjusted our analyses for individuals’ baseline characteristics that acted as confounding factors, using an approach based on a propensity score model.

Individuals’ mobility and balance were measured with the 10-m Timed Walking Test,^62^ the Locomotor Capability Index,^63^^,^^64^ and the Amputee Mobility Predictor.^65^ However, we excluded these measures and other baseline information (eg, residual limb length) from our analyses because they were affected by high rates of missing data (>50%).

Other patient characteristics that could play the role of confounders were retained because the missing data rate, although substantial, was <50%. The Barthel Index was not available on 37% of the hospitalizations, cause of amputation on 35.7%, and Morse Scale on 29.5%. This may have affected the internal validity of some findings about the identification of fall risk factors. For example, whereas the cause of amputation was identified as a significant risk factor for falls with a prosthesis, the Morse Scale was not, although it was employed as a tool for inpatient fall risk assessment. On the other hand, we do not expect that missing values on the covariates of the propensity score model could much affect the adjusted estimates of the association between knee category and falls, because different techniques for missing data management produced only minor changes (data not shown). Among 2429 eligible hospitalizations corresponding to 1235 patients, we included in the analyses 1486 hospitalizations (61%) from 815 patients (66%). The main reasons for not including part of the eligible hospitalizations were difficulty retrieving the paper-based medical records from the archive, the absence of the patient’s consent for treating data for research purposes, and mistakes in the database. Because most of these reasons are related to organizational procedures for data storage, access, and retrieval rather than patient characteristics, we are confident that our estimates are not affected by large biases. However, we do not have data to check the statistical representativeness of the included sample with respect to the whole eligible population.

Most (77.4%) hospitalizations at INAIL Prosthesis Centre were related to individuals with traumatic etiology, whereas it is expected that in the general population, vascular-related conditions are the leading cause of amputation.^1^ Although our dataset encompassed a wide variety of individuals with different clinical profiles, the generalizability of our results to different patient mixes must be verified.

FK exposes people with trans-femoral amputation to a higher risk of falling relative to MPK during rehabilitation training. These findings are based on a wide cohort of individuals, mostly with traumatic amputation. Future research should investigate the relative efficacy of prosthetic devices on individuals with different social and clinical profiles and assess the generalizability of our findings outside the rehabilitation setting.

## Supplementary Material

Supplemental_material_r5_v1_pzab310
