# Supplementary material for: Degree of Safety Against Falls Provided by 4 Different Prosthetic Knee Types in People With Transfemoral Amputation: A Retrospective Observational Study
Source: Phys Ther. 2022 Jan 13;102(4):pzab310. doi: 10.1093/ptj/pzab310 (PMC8994512; doi:10.1093/ptj/pzab310)
Supplement: Supplemental_material_r5_v1_pzab310 [file supplemental_material_r5_v1_pzab310.pdf]

Supplemental material

Table of contents

Prosthetic Knees

Table S1. Grouping of prosthetic knee models according to knee category and frequency on the included hospital stays.

Propensity score

Table S2. Patients’ characteristics before and after PS-weighting

Forms for reporting falls

Figure S1. Form for reporting falls occurred at the Prosthesis Center– part A.

Figure S2. Form for reporting falls occurred at the Prosthesis Center– part B.

Prosthetic knees

Table S1. Grouping of prosthetic knee models according to knee category and frequency on the included hospital stays. Code from the manufacturer, commercial name and model, and name of the manufacturer or assembler are taken from the database of medical devices of the Italian Ministry of Health[1]

Knee category	Code from the manufacturer	Commercial name and model	Manufacturer/Assembler	Frequency (%)
LK	3R32	Ginocchio per moncone lungo - titanio - policentrico, con bloccaggio	Otto Bock Healthcare GmbH	1 (0.07%)
	3R33	3R33 Ginocchio monocentrico in titanio con bloccaggio e deambulante	Otto Bock Healthcare GmbH	219 (15.16%)
	3R40	Ginocchio in lega leggera, monocentrico, con bloccaggio	Otto Bock Healthcare GmbH	18 (1.25%)
	3R62	Pheon	Otto Bock Healthcare GmbH	1 (0.07%)
	3R93	Ginocchio modulare con freno e bloccaggio	Otto Bock Healthcare GmbH	6 (0.42%)
	UNDEFINED CATEGORY LK			6 (0.42%)
AMK	3P23	Prefabbricato di ginocchio - polpaccio Jupa	Otto Bock Healthcare GmbH	24 (1.66%)
	3R36	Ginocchio policentrico Otto Bock Habermann -- Titanio-, con deambulante integrato	Otto Bock Healthcare GmbH	38 (2.63%)
	3R49	Ginocchio con freno automatico - titanio - monocentrico, con deambulante	Otto Bock Healthcare GmbH	49 (3.39%)
	3S80	Modular Sports knee joint	Otto Bock Healthcare GmbH	2 (0.14%)
FK	3R31	Prosedo	Otto Bock Healthcare GmbH	3 (0.21%)
	3R46	Ginocchio per moncone lungo - Titanio -, policentrico, con comando idraulico della fase	Otto Bock Healthcare GmbH	2 (0.14%)

[1] http://www.salute.gov.it/interrogazioneDispositivi/RicercaDispositiviServlet?action=ACTION_MASCHERA

		dinamica		
	3R60	Ginocchio modulare con EBS, policentrico	Otto Bock Healthcare GmbH	63 (4.36%)
	3R60 PRO	Ginocchio polifunzionale con EBS	Otto Bock Healthcare GmbH	
	3R80	Ginocchio monocentrico con idraulica a rotazione e freno automatico	Otto Bock Healthcare GmbH	69 (4.78%)
	3R90	Ginocchio modulare con freno, monocentrico, con deambulante	Otto Bock Healthcare GmbH	1 (0.07%)
	3R92	Ginocchio modulare con freno, monocentrico, con regolazione pneumatica della fase dinamica	Otto Bock Healthcare GmbH	19 (1.31%)
	3R95	Ginocchio monocentrico con comando idraulico della fase dinamica - lega leggera	Otto Bock Healthcare GmbH	2 (0.14%)
	3R106	Ginocchio policentrico con comando pneumatico della fase dinamica	Otto Bock Healthcare GmbH	43 (2.98%)
	N-NK6	Ginocchio idraulico policentrico a 6 assi	Nabco Company Welfare Products DPT	112 (7.75%)
	N-NK6B	Ginocchio idraulico policentrico a 6 assi con bloccaggio selettivo	Nabco Company Welfare Products DPT	
	MKN01360	Mauch Knee SNS	Ossur	29 (2.01%)
	MKN01361	Mauch Knee SNS Low Res	Ossur	
	MKN01362	Mauch Knee Swing Only	Ossur	
	MKN01660	Mauch Knee Plus SNS	Ossur	
	MKN01661	Mauch Knee Plus SNS Low Res	Ossur	
	MKN01662	Mauch Knee Plus Swing	Ossur	
	MKN01665	Mauch Knee Plus SNS Through Hole	Ossur	
	MKN01666	Mauch Knee Plus SNS Low Res Through Hole	Ossur	
	MKN01667	Mauch Knee Plus Swing only Through Hole	Ossur	
	MKN01663	Mauch Knee Plus con cilindro a	Ossur	

		resistenza regolare, foro passante		
	MKN01664	Mauch Knee Plus con cilindro a bassa resistenza foro passante	Ossur	
	2000	Total Knee 2000	Ossur	96 (6.64%)
	2000-KE	Total Knee 2000 disarticolazione del ginocchio con giunto per invasature	Ossur	
	2000-OS	Total Knee 2000 transfemorale con giunto piramidale maschio	Ossur	
	2100	Total Knee 2100	Ossur	22 (1.52%)
	2100-KE	Total Knee 2100 disarticolazione del ginocchio con giunto per invasature	Ossur	
	TGK 5PS10	Ginocchio carbonio policentrico pneurmatico	Teh Lin Prosthetic & Orthopaedic Inc.	8 (0.55%)
	MK30, MHPYR	Mercury Hi Activity	Blatchford Products LTD	3 (0.21%)
	KX06	KX06	Blatchford Products LTD	17 (1.18%)
	CKN10000	Cheetah Knee	Ossur	1 (0.07%)
	UNDEFINED CATEGORY FK			8 (0.55%)
MPK	3C88-3	C-Leg 4	Otto Bock Healthcare GmbH	388 (26.85%)
	3C88-1	C-Leg	Otto Bock Healthcare GmbH	
	3R80	Tubo modulare C-Leg	Otto Bock Healthcare GmbH	
	3C98-1	C-Leg	Otto Bock Healthcare GmbH	
	3C86-1	C-Leg compact	Otto Bock Healthcare GmbH	
	3C96-1	C-Leg Compact	Otto Bock Healthcare GmbH	
	3C98-2	Ginocchio elettronico C-Leg	Otto Bock Healthcare GmbH	
	3R82	Tubo modulare C-Leg	Otto Bock Healthcare GmbH	
	3C88-2	C-Leg	Otto Bock Healthcare GmbH	
	3C98-3	Ginocchio elettronico C-Leg	Otto Bock Healthcare GmbH	
	3B1	Ginocchio elettronico Genium Bionic	Otto Bock Healthcare GmbH	77 (5.3%)
	3B5	Genium X3	Otto Bock Healthcare GmbH	13 (0.9%)
	3C60	Kenevo	Otto Bock Healthcare GmbH	6 (0.42%)

	RKN100003; RKN100103; RKN1XXXX; RKN3XXXX	Rheo Knee Kit	Ossur	23 (1.59%)
	RKN12000; RKN120003; RKN120101; RKN120103	Rheo Knee II	Ossur	
	N-NIC311	Hybrid - Ginocchio elettronico idraulico/pneumatico	Nabtesco Corporation	23 (1.59%)
	UNDEFINED CATEGORY MPK			53 (3.67%)

LK= locked knee; AMK= articulating mechanical knee; FK= fluid-controlled knee; MPK= microprocessor-controlled knee.

Propensity score

Table S2. Patients' characteristics before and after PS-weighting. Population mean and standard deviation (sd); unweighted mean per prosthetic knee category; unweighted MES; PS-weighted mean per prosthetic knee category; PS-weighted MES. Patients' risk factors for falls are highlighted in grey.

	**Mean**	**Sd**		**Unweighted mean**				**MES**		**PS-weighted mean**				**MES**
**variable**	**Population**			**LK**	**AMK**	**FK**	**MPK**			**LK**	**AMK**	**FK**	**MPK**	
Age	58.159	14.695		70.854	60.723	54.822	55.346	0.364		70.48	60.24	58.14	53.37	0.327
Sex (Female)	0.090	0.286		0.235	0.124	0.098	0.014	0.230		0.19	0.11	0.1	0.01	0.173
Weight (kg)	77.313	14.314		72.007	74.476	78.094	79.222	0.189		72.49	75.45	78.65	78.83	0.167
Height (m)	1.711	0.078		1.670	1.705	1.718	1.723	0.212		1.67	1.7	1.71	1.73	0.218
Reason for rehabilitation training (first prosthetic provision)	0.222	0.415		0.454	0.205	0.338	0.028	0.337		0.24	0.22	0.22	0.08	0.102
Goal of the rehabilitation training														
Walking with one cane or crutch	0.338	0.473		0.427	0.518	0.420	0.187	0.265		0.44	0.57	0.41	0.18	0.298
Walking with two crutches	0.101	0.301		0.294	0.064	0.099	0.014	0.266		0.3	0.07	0.09	0.02	0.267
Unassisted gait	0.521	0.500		0.097	0.418	0.466	0.797	0.430		0.13	0.36	0.49	0.8	0.434
Walking with a walker	0.039	0.194		0.181	0.000	0.015	0.002	0.313		0.14	0	0.01	0	0.258
Length of stay (**days**)	**21.71**	**17.38**		**26.93**	**20.64**	**23.87**	**17.93**	**0.176**		25.98	22.59	21.99	18.45	0.125
Third-party payer														
ASL	0.216	0.412		0.446	0.407	0.279	0.017	0.415		0.27	0.43	0.23	0.05	0.265
INAIL	0.739	0.439		0.438	0.558	0.667	0.971	0.447		0.65	0.51	0.74	0.93	0.290
Private	0.045	0.208		0.116	0.035	0.054	0.012	0.147		0.08	0.06	0.04	0.02	0.101

Amputation cause														
Cancer	0.034	0.180		0.051	0.043	0.053	0.003	0.106		0.04	0.03	0.04	0	0.061
Congenital	0.013	0.111		0.006	0.057	0.003	0.000	0.166		0.02	0.01	0	0	0.069
Infectious	0.026	0.160		0.034	0.057	0.036	0.003	0.112		0.02	0.02	0.03	0	0.047
Traumatic	0.774	0.418		0.382	0.714	0.794	0.988	0.410		0.73	0.75	0.78	0.94	0.146
Vascular	0.154	0.361		0.528	0.129	0.114	0.006	0.407		0.19	0.18	0.16	0.05	0.118
Amputation side (left)	0.528	0.499		0.432	0.482	0.551	0.565	0.101		0.43	0.48	0.56	0.55	0.107
Time from amputation (months)	204.75	207.95		154.60	328.38	139.40	264.9	0.360		207.54	225.04	204.33	242.37	0.073
Number of comorbidities	5.315	2.210		6.861	5.248	5.263	4.679	0.260		6.53	5.23	5.23	4.62	0.236
Number of drugs	3.519	4.011		6.625	3.708	3.323	2.403	0.287		5.54	3.91	3.32	2.23	0.243
Use of antipsychotics	0.022	0.145		0.032	0.009	0.038	0.007	0.094		0.03	0.01	0.04	0.01	0.095
Use of antidepressants	0.086	0.281		0.159	0.035	0.088	0.062	0.134		0.09	0.05	0.07	0.06	0.068
Use of benzodiazepines	0.030	0.171		0.048	0.035	0.028	0.024	0.045		0.03	0.06	0.02	0.03	0.064
Use of loop diuretics	0.058	0.234		0.203	0.035	0.036	0.019	0.245		0.16	0.03	0.04	0.02	0.214
Use of beta-blocking agents	0.157	0.364		0.295	0.133	0.133	0.129	0.148		0.26	0.11	0.14	0.12	0.142
Use of opioids	0.056	0.230		0.064	0.053	0.076	0.038	0.054		0.05	0.09	0.06	0.03	0.073
Use of antiepileptics	0.098	0.297		0.159	0.035	0.147	0.046	0.189		0.13	0.06	0.11	0.05	0.101
Morse Scale	39.132	14.359		47.365	40.309	38.964	36.005	0.221		45.25	38.95	38.76	36.03	0.170
Barthel Index	92.641	12.162		80.647	92.082	91.917	99.358	0.411		85.96	92.78	94.13	99.32	0.308

Sd= standard deviation; MES= mean effect size, i.e. mean of the absolute standardized difference between prosthetic group and population means; PS=propensity score.

Forms for reporting falls

Figure S1. Form for reporting falls occurred at the Prosthesis Center– part A. Filled in by anyone of the staff of the Prosthesis Center.

INAIL Centro Protesi	MODULO SEGNALAZIONE CADUTE PARTE A	rif. Mod. 125
		ver. 00
		del 01/08/11

Identificazione del soggetto

□Paziente □ Degente □ Esterno	Nome	Cognome
	Data di nascita	Luogo di nascita
□ Visitatore	Sesso: □ Maschio	□ Femmina
Data caduta	Ora caduta	
Tipo di menomazione		
Il paziente indossava protesi/ortesi	□ Si	□ No
□ Quasi evento	Evento:	□ Con danno apparente<br>□ Nessun danno apparente

Luogo dell'evento

□ Camera	□ Corridoio	□ Bagno
□ Scale	□ Esterno	□ Sala d'attesa
□ Palestra	□ Ambulatorio	□ Officina
□ Fisioterapia	□ D.H.	□ Altro (specificare)

Identificativo del luogo (es. corridoio 1° Piano)_____________ _______________

Modalità della caduta

□ Dal letto	□ Trasferimenti	□ Dalla sedia a rotelle
□ Spostamenti con stampelle	□ Durante il cammino	□ Dalla posizione eretta fermo
□ Dalla sedia	□ Altre situazioni:	

Dinamica dell'evento

....

....

....

....

...........,

Segnalatore

Nome	Cognome	Reparto / Servizio

□ Medico □Infermiere □ OTA/OSS □ Tecnico

□ Fisioterapista □ Amministrativo □ Altro________

Avvisato medico: □ NO □ SI

Dott.____________________________

Data	Firma

Figure S2. Form for reporting falls occurred at the Prosthesis Center– part B. Filled in by a member of the Clinical Risk Group.

INAIL Centro Protesi	MODULO SEGNALAZIONE CADUTE PARTE B	rif. Mod. 125
		ver. 00
		del 01/08/11

**Identificazione dell'evento**	
Nome	Cognome
Data Caduta____________________	Ora caduta______________________

Fornitura □ 1° fornitura □ Successiva

Tipo di protesi/ortesi...

Tipo di danno

□ Trauma cranico □ Fratture □ Distorsioni

□ Contusioni □ Ferite □ Escoriazioni

□ Ematomi □ Nessun danno

□Altro (specificare)____

Fattori che possono aver contribuito all'evento

Condizioni del paziente	□ Cadute pregresse<br>□ Terapie farmacologiche<br>□ Condizioni cliniche<br>□ Alterazioni mobilità<br>□ Altro(specificare)	□ Stato mentale<br>□ Abbigliamento / calzature<br>□ Problema evacuazione / diuresi<br>Note
Condizioni organizzative	□ Carenza personale<br>□ Non corretta applicazione step addestrativo / riabilitativo<br>□ Altro (specificare)...	□ Comunicazione difficoltosa
Condizioni ambientali (pavimento bagnato ecc)		
Breve descrizione dell'accaduto		

Compilatore

Nome	Cognome	Unità Rischio Clinico
Data	Firma	
